# Identification of the Epileptogenic Zone from Stereo-EEG Signals: A Connectivity-Graph Theory Approach

**DOI:** 10.3389/fneur.2013.00175

**Published:** 2013-11-06

**Authors:** Ferruccio Panzica, Giulia Varotto, Fabio Rotondi, Roberto Spreafico, Silvana Franceschetti

**Affiliations:** ^1^Neurophysiology and Diagnostic Epileptology Operative Unit, “C. Besta” Neurological Institute IRCCS Foundation, Milan, Italy; ^2^Department of Informatics, Bioengineering, Robotics, System Engineering (DIBRIS), University of Genova, Genova, Italy; ^3^Clinical Epileptology and Experimental Neurophysiology Operative Unit, “C. Besta” Neurological Institute IRCCS Foundation, Milan, Italy

**Keywords:** focal drug-resistant epilepsy, epilepsy surgery, stereo-EEG, connectivity, causality, epileptogenic zone, graph theory, brain networks

## Abstract

In the context of focal drug-resistant epilepsies, the surgical resection of the epileptogenic zone (EZ), the cortical region responsible for the onset, early seizures organization, and propagation, may be the only therapeutic option for reducing or suppressing seizures. The rather high rate of failure in epilepsy surgery of extra-temporal epilepsies highlights that the precise identification of the EZ, mandatory objective to achieve seizure freedom, is still an unsolved problem that requires more sophisticated methods of investigation. Despite the wide range of non-invasive investigations, intracranial stereo-EEG (SEEG) recordings still represent, in many patients, the gold standard for the EZ identification. In this contest, the EZ localization is still based on visual analysis of SEEG, inevitably affected by the drawback of subjectivity and strongly time-consuming. Over the last years, considerable efforts have been made to develop advanced signal analysis techniques able to improve the identification of the EZ. Particular attention has been paid to those methods aimed at quantifying and characterizing the interactions and causal relationships between neuronal populations, since is nowadays well assumed that epileptic phenomena are associated with abnormal changes in brain synchronization mechanisms, and initial evidence has shown the suitability of this approach for the EZ localization. The aim of this review is to provide an overview of the different EEG signal processing methods applied to study connectivity between distinct brain cortical regions, namely in focal epilepsies. In addition, with the aim of localizing the EZ, the approach based on graph theory will be described, since the study of the topological properties of the networks has strongly improved the study of brain connectivity mechanisms.

## Introduction

Focal epilepsies, in which the seizures originate from a region limited to a part of one cerebral hemisphere (1), are common and account for more than 50% of all epilepsies (2). Despite the great improvement in pharmacological research, approximately 30% of patients with focal epilepsies experience seizures that are resistant to anti-epileptic drugs (AEDs) (3). In these patients the surgical resection of the epileptogenic zone (EZ), the cortical region responsible for the onset, early organization, and propagation of seizures, may be the only way to suppress or reduce seizures. The EZ represents the minimum amount of cortex that must be resected (inactivated or completely disconnected) in order to achieve seizure freedom (4).

The EZ can sometimes be adequately localized by means of non-invasive investigations including clinical neurological examination, detailed description of ictal signs and symptoms, Magnetic Resonance Imaging (MRI), Positron Emission Tomography (PET), and interictal and ictal scalp video-EEG recordings. However, when the region of seizure onset cannot be precisely identified non-invasively, or when the obtained information is insufficient to exclude the involvement of “eloquent cortical areas” in seizure generation, invasive electrophysiological exploration can be performed from brain structures in order to evaluate their potential involvement in the epileptogenic process using stereo-EEG (SEEG) recordings by means of the stereotactic surgical placement of intracranial electrodes (5, 6), associated with video recordings, with the aim of planning a tailored resection based on individual anatomical and electroclinical characteristics. After invasive recordings, the EZ is currently identified by visually inspecting the video-SEEG recordings, a procedure that requires the involvement of specifically trained neurophysiologists and is inevitably affected by the drawback of subjectivity. Moreover, the rather high rate of failure in epilepsy surgery of extra-temporal epilepsies (7) underlines that the precise identification of the EZ is still an unsolved problem that requires more sophisticated methods of investigation.

Over the last years, great efforts have been made to develop and implement advanced signal analysis techniques able to improve the identification of the EZ. Particular attention has been paid to those methods aimed at quantifying and characterizing the interactions and causal relationships between neuronal populations, since is nowadays well assumed that epileptic phenomena are associated with abnormal changes in brain synchronization mechanisms, and a number of studies have shown that seizures are associated with the abnormal synchronization of distant structures (8–10). The aim of this review is to provide an overview of the different intracranial EEG signal processing methods used to identify the EZ, with particular attention being given to the methods aimed at characterizing the brain connectivity. In addition, the approach based on graph theory will be described, since the study of the topological properties of the networks has strongly improved the study of brain connectivity mechanisms.

## Brain Connectivity

It is well known that human brain can be considered as a hierarchical complex structure, and that most of the brain functions are based on interactions among neuronal assemblies distributed within and across distinct cerebral regions (11). The concept of brain connectivity can be subdivided into three main categories (12–15) (a) anatomical (or structural) connectivity, which indicates the set of physical or structural connections linking neurons; (b) functional connectivity, defined as the temporal correlation expressed in terms of the statistical dependence between spatially remote neuronal populations; and (c) effective connectivity, which refers to the influence that one neural system exerts over another, thus taking into account the direction of the information flow from one region toward another.

Several techniques have been developed to study interactions in time and/or frequency domain, and in both linear and non-linear contexts. An important distinction between methods aimed at estimating connectivity is that between bivariate and multivariate measures (16, 17). Bivariate methods are able to evaluate the existence of interactions by considering only couple of signals; this can lead to the identification of spurious connections, as these methods do not allow to distinguish between direct and indirect relationships. Many recent papers have extensively described the advantages and disadvantages of bivariate measures, both in the field of functional and effective connectivity (18–21). On the contrary, multivariate measures allow the exploration of the whole network, by considering the entire set of signals in the same model.

A second important point is that functional connectivity, although useful in characterizing brain networks, does not provide any information concerning the direction of interactions, an issue very important in the field of EZ identification. To overcome these limitations, directed connectivity measures have been developed with the aim of estimating effective causality.

A large group of effective connectivity measures are based on the concept of Granger causality (22), initially defined for bivariate processes *x*(*t*) and *y*(*t*): *x*(*t*) Granger causes *y*(*t*) if the knowledge of the past of both *x*(*t*) and *y*(*t*) reduces the variance of the prediction error of *y*(*t*), in comparison with the knowledge of the past of *y*(*t*) alone. The lack of reciprocity makes it possible to evaluate the direction of the information flow between the signals. Granger’s definition of causality can be easily implemented by means of autoregressive (AR) models, and extended to the case of multivariate systems (23, 24).

Several directed connectivity measures have been developed on the basis of the concept of Granger causality derived from the multivariate autoregressive (MVAR) modeling. Among them, directed transfer function (DTF) (25) and partial directed coherence (PDC) (26) are effective connectivity measures based on MVAR coefficients transformed into the frequency domain. PDC is particularly interesting because of its ability to distinguish direct and indirect causality flows. Particular care must be employed in using PDC, DTF, and other Granger causality-based measures of effective connectivity since they require that the signals are stationary. In epilepsy research this condition is often not matched, especially for ictal discharges. Recently, time varying versions of effective connectivity estimation methods, such as the ADTF (Adaptive DTF) (27, 28), have been proposed and successfully applied on intracranial signals of patients suffering from refractory epilepsy (29).

The main limitations of these approaches, however, are that the MVAR modeling assumes the hypothesis of the linearity of the underlying process and that it does not allow to study systems with high number of channels, because the number of parameters to be estimated must be lower than the number of samples (30).

To overcome these drawbacks non-linear, mainly bivariate, approaches have been developed (16, 31). Among these, one method largely applied in the field of intracranial EEG is the non-linear regression index *h*^2^, introduced by Pijn and Lopes da Silva (32) to analyze EEG signals, and subsequently extended to SEEG by Wendling et al. (33). This approach has mainly been used to investigate the connectivity between the temporal neocortex and limbic structures in patients with temporal lobe epilepsy (TLE) (34, 35).

## Graph Analysis

The study of the functional or effective connectivity by means of linear or non-linear methods may not be enough to grasp the full complexity of the brain due to the huge amount of data obtained. Indeed, evaluation of interconnections between all possible pairs of EEG electrodes or SEEG contacts in a particular frequency band will produce huge matrices of correlation data, difficult to interpret and to handle statistically. To overcome these difficulties an approach based on graph theory (36), derived from the theory of complex networks, could provide useful measures to characterize the topological properties and the functional organization of the brain networks involved both in normal brain functioning and diseases.

According to this approach, the brain is represented as a graph consisting of a set of nodes, or vertices (the EEG electrodes, SEEG contacts, MEG sensors, fMRI voxels, …) and edges, or links, indicating the presence of an interaction between pairs of nodes. Edges can be directed, as in the case of effective connectivity methods, putting in evidence that the activity of one node may depend on the other and not vice-versa, or undirected when direction was not evaluated (e.g., functional connectivity approach). If the value of the edges are taken into account the graph or network is called weighted, otherwise unweighted.

The procedure to study (intracranial) EEG using a graph theory approach usually includes the following steps. After estimating brain connectivity, an association matrix is generated whose elements are the estimated values between each pair of nodes. A binary or weighted adjacency matrix of undirected (symmetric) or directed graphs is then produced by applying a threshold to each element of the associated matrix. The choice of this threshold is a crucial issue, since it determines the number of the existing connections; therefore different thresholds will produce different graphs, and may affect several network indices (37).

Graphs can be characterized by various measures, each aimed at describing different topological properties of the network, allowing to characterize the network organization (i.e., random, regular, small-world; see (38) for a definition of these topological properties) and/or the global or local properties of each node of the network.

Since the main aim of this review is to characterize specific properties of the EZ, we will focus on the indexes representing node’s specific properties. For a more detailed description of these indexes, including their mathematical representation, and other graph measures, see (12, 31, 39, 40).

Graph indexes can be broadly divided into three main categories – measures of centrality, segregation, integration – according to the network properties that they better describe.

Centrality measures the structural and functional importance (40) of each node with respect to the rest of the network; it is one of the main measures used to identify the hubs of a network, that is, nodes that interact with many other regions, playing a key role in functional integration.

Main measures of centrality are:

- Degree: the total number of edges connected to a node. In directed networks, is possible to distinguish between in-degree, the number of inward links, and out-degree, the number of outward edges. The mean degree of a graph is the average degree over all vertices.
- Density: the actual number of edges divided by the total number of possible edges in a graph.
- Betweenness centrality: the ratio between the number of shortest paths (defined as the smallest number of edges between two nodes) passing through a specific node and the total number of shortest paths in the network. It accounts for the importance of a node in facilitating interactions between other nodes in a network.
- Eigenvector centrality. This index also measures the importance of a node on the basis of the number (and strength) of links to other nodes, but it takes also into account if the node has strong connections with others having themselves a central position within the network.

Segregation refers to the existence of specialized brain areas where specific processes occur such as those responding to specific sensory inputs. Main measures of segregation are:

- Clustering coefficient: it measures the degree to which nodes in a graph tend to cluster together and is defined as the fraction of triangles around a node over the total number of possible triangles; it represents the fraction of a node’s neighbors that are also neighbors of each other (38).
- Modularity is a more sophisticated measure able to detect and quantify the presence of segregated activity (40). A module represents a cluster of densely interconnected groups of nodes. In order to partition the graph into modules, it is necessary to find the optimal community structure, defined as a subdivision of the network into non-overlapping groups of nodes in a way that maximizes the number of within-group edges, and minimizes the number of between-group edges. Once a graph has been partitioned into modules, other two indexes can be defined.
- Participation coefficient: measure of diversity of inter-modular connections of individual nodes.
- Within degree: within-module version of degree centrality.

Integration refers to the ability to combine specialized processes distributed into different brain regions. Main measures of integration include:

- Shortest Path length: it measures the shortest path (or distance) between each pair of nodes, where the path is a sequence of distinct nodes and edges representing potential routes of information flow between pairs of regions. The path length is infinite in case of disconnected pairs of nodes. The average shortest path length between all pairs of nodes is called the characteristic path length of the network and is the most commonly used measure of functional integration.
- Efficiency: the efficiency of a connection between two nodes is defined as the inverse of the path length. Global efficiency is the average of all the inverse shortest path lengths (12), while local efficiency measures the efficiency of the connections between the neighbors of a node when the node itself is removed. The global efficiency may be computed on disconnected networks, since paths between disconnected nodes have zero efficiency.

Graph theory has been widely used to investigate epilepsy. It is commonly assumed that epileptic seizures are due to excessive neuronal firing and synchronization, but the underlying mechanisms are still not clear. For a recent review in this field see (41). The first indication that network analysis might be helpful in understanding epilepsy came from the simulation study of Netoff and others (42) on models of hippocampal networks. The authors showed that changes in the network topology could induce transitions from normal behavior to seizure. After this study, several works investigated the global network topology in patients with different types of epilepsy. Ponten and others (43), studying EEG recordings reported that absences are characterized by an increase in synchronization and that the functional network topology changed toward a more ordered pattern when compared to pre-ictal network configuration. Chavez and others (44) analyzed the connectivity topology of brain networks extracted from MEG signals of patients with absence seizures recorded at rest and found the brain networks of patients to display a richer connectivity with a clear modular structure with respect to controls. van Dellen and others (45), investigating the effects of on-going TLE, showed that functional connectivity was lower in patients with longer TLE history, and that longer TLE duration was correlated with more random network configuration.

Bartolomei and colleagues (46) investigated the topological properties of the epileptogenic networks of 11 patients presenting drug-resistant mesial temporal lobe epilepsy (MTLE) using as control group 8 patients with neocortical epilepsy. They found that the network organization of the interictal SEEG activity in both groups were characterized by a small-world model; network topology was however more altered in the MTLE group with respect to non-MTLE patients and corresponded to a more regular (less random) configuration, interpretable as an increase of local and a decrease of long distance connections. The authors suggested that these changes in topology could be a potential biomarker of epileptogenic network.

Overall, these studies indicate that a complex network approach may offer new methodologies not only for improving our understanding on the basic mechanisms of epilepsy, but also could potentially yield quantitative measures to be used as indicators or biomarkers for early detection and diagnosis of epilepsy, the assessment of its course, and for the evaluation of the effect and the efficacy of treatments.

## EZ Localization

So far, only a few studies have investigated network alterations in relation to the EZ using graph theory in patients undergoing surgery for intractable epilepsies. Wilke et al. (29) used DTF and betweenness centrality to identify critical network nodes during ictal and interictal electrocorticogram recordings. They found that the betweenness centrality correlated with the location of the resected cortical regions in patients who were seizure-free following epilepsy surgery. Furthermore, the better outcome was associated with the resection of brain regions showing the highest betweenness centrality. This finding suggested that critical highly interconnected nodes played a crucial role in seizure onset and spreading. Importantly, the authors observed patterns of altered network interactions (mainly in the gamma band) not only during seizures, but also during interictal spike activity and random non-ictal periods. This result supported the hypothesis that functional brain networks of patients with focal epilepsies may be altered even during the seizure-free periods (47).

In a very recent paper, van Mierlo et al. (48) studied the temporal changes in effective connectivity during the first 20 s of ictal rhythmic intracranial EEG activity in the 3–20 Hz band, in eight patients suffering from focal intractable epilepsy who underwent successful resective surgery. In all the patients, the authors found that the SEEG contact showing maximal ictal out-degree was among those visually classified by clinicians as belonging to the seizure onset zone (49), and it was always included within the resected brain region. Furthermore, the patient-specific connectivity patterns were consistent over the majority of seizures, suggesting that most of the seizures originated from the same area and that the same driving hubs play a leading role into the epileptogenic network.

We (50) studied the changes in connectivity patterns in 10 patients with Taylor-type focal cortical dysplasia (type II FCD) under interictal, pre-ictal, and ictal conditions to characterize the network properties of the SEEG signals recorded from inside the dysplasia and distinguish them from those of other regions involved in ictal activity or not. We selected this type of focal epilepsy in order to validate the appropriateness of our approach because this type of dysplasia is intrinsically epileptogenic (51), the EZ normally overlaps the dysplasia. We applied the PDC to study the effective connectivity in the frequency domain, and characterized the epileptogenic network by means of out-density and betweenness centrality measures.

Our findings indicated that the region inside the dysplasia was characterized by abnormal out-going connectivity in comparison with the other examined areas. The main changes were observed in the gamma band, between 30 and 70 Hz. This specific connectivity pattern was also present in the interictal state, temporally distant from seizures several minutes, and even in the absence of obvious epileptiform activity. This suggests that key information that can help clinicians in localizing the EZ are included in the interictal SEEG activity and that effective connectivity and graph theory indexes are useful tools capable of disclosing them.

As a further step, we compared the differences in connectivity patterns occurring between the interictal, pre-ictal, and ictal conditions. The time course of the increase in synchronization of SEEG activity recorded from the dysplasia and other regions showing epileptiform activity was different; indeed a significant increase in out-going synchronization between the contacts within the EZ and from the EZ toward other areas occurred during the pre-ictal period with respect to the interictal state, whereas an increase of interactions in regions outside the dysplasia but involved in the ictal events occurred later on, at the time of seizure onset, marking the significant differences in connectivity between the ictal and pre-ictal state.

Altogether, our findings suggest that, in patients with type II FCD, the region inside the dysplasia plays a leading role in generating and propagating ictal EEG activity, and in recruiting other distant areas to become involved in the seizure; this area acts as an abnormal hub of the epileptic network that originates and sustains the seizures. The cortical areas outside the dysplasia involved in the SEEG ictal activity, act essentially as “secondary” generators of synchronous activity. The leading role of the dysplasia may account for the good post-surgical outcome of patients with type II FCD because the resection of dysplastic tissue removes the entire EZ responsible for seizure onset.

## Conclusion

Accurate localization of the EZ is the goal of pre-surgical work-up in patients with drug-resistant focal epilepsies. Over the last years, much research has been dedicated to develop advanced signal processing techniques with the aim of studying the topological properties of functional brain networks in patients with epilepsy. Under the hypothesis that the brain tissue associated with the EZ is differently connected within an epileptic network compared to other regions, in the last years few groups have begun to investigate how the EZ gives rise to network alterations in patients with focal epilepsy, using an approach based on brain connectivity and graph theory. These studies indicate that this network-based approach may add new and valuable information, providing quantitative measures useful for localizing the EZ or for greatly reducing the number of contacts.

Interesting and promising is the finding that network alterations related to EZ are present and could be detected also during EEG interictal periods, since it suggests that, in the future, network-based algorithms could potentially be used to reduce the need for long-term monitoring in patients with drug-resistant focal epilepsy.

More studies in larger and possibly multicentre patient populations that include patients with drug-resistant focal epilepsy of different (or unknown) etiology, however, are expected to clarify which network measures work best in localizing the epileptogenic network and to validate them.

## Conflict of Interest Statement

The authors declare that the research was conducted in the absence of any commercial or financial relationships that could be construed as a potential conflict of interest.
